# Effects of CO_2_ Curing on the Properties of Pervious Concrete in Different Paste–Aggregate Ratios

**DOI:** 10.3390/ma16134581

**Published:** 2023-06-25

**Authors:** Mingfang Ba, Siyi Fang, Wei Cheng, Yawen Zhao

**Affiliations:** School of Civil and Environmental Engineering, Ningbo University, No. 818 Fenghua Road, Ningbo 315211, China18758360316@163.com (Y.Z.)

**Keywords:** pervious concrete, early CO_2_ curing, paste–aggregate ratio, mechanical properties, porosity

## Abstract

To improve the comprehensive performance of pervious concrete, the properties of pervious concrete in different paste–aggregate ratios were subjected to both early CO_2_ curing and uncarbonated curing conditions. The mechanical properties, water permeability, porosity, and chemical composition of pervious concrete under two curing conditions were investigated and compared. The effects of CO_2_ curing on the properties of pervious concrete with different paste–aggregate ratios were derived. Through mechanical experiments, it was revealed that early CO_2_ curing can enhance the mechanical strength of pervious concrete by about 15–18%. Meanwhile, with the increase in the paste–aggregate ratio, the improvement effect induced by early CO_2_ curing became more significant. The water resistance of carbonated concrete was not significantly reduced. And with the increase in the paste–aggregate ratio, the carbonation degree of pervious concrete was reduced; the differences in porosity and water resistance became less significant when the paste–aggregate ratio exceeded 0.39. Micro-structural analysis shows that the early CO_2_ curing reduced both total porosity and the volume of micropores with a pore diameter of less than 40 nm, while it increased the volume of pores with a diameter of more than 40 nm. This is also the main reason that the strength of pervious concrete under early CO_2_ curing is higher than that without CO_2_ curing. The effect of varying paste–aggregate ratio and curing methods adds to the limited knowledge of the performance of pervious concrete.

## 1. Introduction

As a green infrastructure solution for urban areas, pervious concrete has received significant attention in recent years as the permeable pavement material used by many cities worldwide [1,2]. Especially in China, the application of pervious concrete has become a sustainable solution to the increasingly severe urban rainwater management and control problems, as it can quickly immerse rainwater into the ground during floods, thereby alleviating the problem of urban waterlogging. At the same time, it is also a major material that will be used in urban construction in the future [3]. As a gap-graded material, pervious concrete consists of interconnected aggregates and connected capillary pores [4]. The aggregate gradation for the pervious concrete generally includes single-sized coarse aggregates/binary mixture of coarse aggregates with the optimal amount of cement to coat and bind aggregates together [5]. The cementation material wrapped on the aggregate surface acts as the cementation layer, forming a honeycomb porous material with skeleton pore structure, leading to many pores in pervious concrete [6,7,8]. Therefore, the water permeability and strength of pervious concrete are often incompatible due to the large number of voids and larger pores.

Carbonization curing, as a new green curing technology, can not only improve the performance of cement-based materials, but also achieve the carbon dioxide capture and reduction in the current global greenhouse effect [9,10]. Through carbonization curing, carbon dioxide can be captured to form stable insoluble calcium carbonate in the early hydration stages. It is generally known that carbonization curing significantly improves the early strength and compactness of cement-based materials, as well as the long-term performance of cement-based materials in complex environments [11,12,13]. This is of great significance for improving the comprehensive performance of pervious concrete.

CO_2_ curing, as a sustainable method, has been studied by researchers both domestically and internationally to improve the performance of pervious concrete. Tian et al. [14] believes that the appropriate early curing conditions are beneficial for refining the porosity characteristics. Chen and Gao [15] found that CO_2_ curing could improve the compressive strength of pervious concrete, especially at early ages. In addition, CO_2_ curing could significantly improve the freeze−thaw and leaching resistance of pervious concrete. Macmaster [16] proposed that the carbonation reaction between carbon dioxide and calcium compounds could permanently fix carbon dioxide in a thermodynamically stable slurry. Tanaka et al. [17] also proved that accelerated carbonation improved the strength and durability of the surface pervious concrete framework. Hasegawa [18] believes that early CO_2_ curing can effectively improve the strength of pervious concrete. Chen [19] found that appropriate CO_2_ curing can significantly improve the early compressive strength of pervious concrete. Compared to ordinary concrete, the carbonation degree of cement stone inside pervious concrete is higher, and the internal and external carbonation is more uniform. Fang et al. [20] improved the performance of aggregates and concrete by strengthening steel slag aggregates and steel slag pervious concrete through carbonization. The results showed that steel slag aggregates generate calcium carbonate to fill the pores during CO_2_ curing, thereby improving the density of the aggregates. Wu and Xu [21] explored the appropriate substitution amounts of carbonated aggregates in pervious concrete. Liu [22] greatly improved the carbon fixation ability of pervious concrete by adding steel slag to it. In addition, research has also been conducted on other curing methods for pervious concrete both domestically and internationally. Zhang et al. [23] conducted research on the early curing of pervious concrete and proposed that the use of covered film moisturizing curing can significantly improve the strength of pervious concrete at 7 and 28 days. Kevern et al. [24] studied the internal curing method of pervious concrete and determined that internal curing by saturating the aggregate with water can significantly improve the compressive strength of pervious concrete while reducing its dry shrinkage deformation.

In summary, early CO_2_ curing can improve the strength of pervious concrete, showing good carbon fixation ability. However, comprehensive reports on the effects of CO_2_ curing on the properties of pervious concrete in different paste–aggregate ratios are still limited. Based on this, this paper aims to study the influence of paste–aggregate ratio on the strength, permeability, porosity, and composition of pervious concrete with and without early CO_2_ curing treatment, to analyze how early CO_2_ curing could influence the performance of pervious concrete in different paste–aggregate ratios. The perpetual fixation of carbon dioxide in pervious concrete contributes to the sustainable development of the construction. Through this study, it is expected to provide more theoretical and technical support for the preparation of high-performance pervious concrete and ecological environment construction while capturing more carbon dioxide at the same time.

## 2. Material and Experiments

### 2.1. Raw Materials

The cement was ordinary Portland cement (P·O42.5) created by Hailuo Cement Co., Ltd., in Anqing, China. Its chemical composition and performance parameters are shown in Table 1 and Table 2, respectively. Polycarboxylate super-plasticizer was provided by Kezhijie New Materials Co., Ltd., in Xiamen, China with a water-reducing rate of 25%. The particle size of the crushed stone aggregate is 4.75–9.5 mm, and the gradation is in single grain. The physical index of aggregate is listed in Table 3. Deionized water was used for mixing various mixtures. The pervious concrete of this study satisfies all the technical specifications for pervious cement concrete pavements listed in Chinese industrial standard CJJ/T135-2009 [25].

### 2.2. Sample Preparation and Experiments

The pervious concrete design method based on the optimal paste–aggregate ratio [26] is used to design the mix proportion of pervious concrete with water–binder ratio of 0.2. The corresponding paste–aggregate ratio is adjusted into five scales, including 0.30, 0.35, 0.39, 0.43 and 0.45, respectively. Detailed information of the mix proportion is shown in Table 4.

Based on the mix proportions in Table 4, a series of pervious concretes were prepared for tests. Cubic specimens with side lengths of 100 mm were prepared for compressive experiments. Specimens, 100 mm × 100 mm × 400 mm in size, were prepared for flexural strength experiments. Cylindrical specimens Φ100 mm × 50 mm in size were prepared for water permeability coefficient experiments. After removing the mold, the specimens in each proportion were cured with and without the early CO_2_ curing method, respectively.

The uncarbonated curing method was used to cover the specimens with a layer of thin membrane, to prevent it from coming into contact with carbon dioxide in the air. The early CO_2_ curing was carried out under accelerated carbonation conditions referring to the Testing Methods for the Long-term Performance and Durability of Ordinary Concrete (GB/T 50082-2009) [27]. During the test, the carbon dioxide concentration in the chamber was kept at (20 ± 3)%, the relative humidity was controlled at (70 ± 5)%, and the temperature was controlled at (20 ± 2) °C, and no specific pressure was maintained.

**Figure 1:**
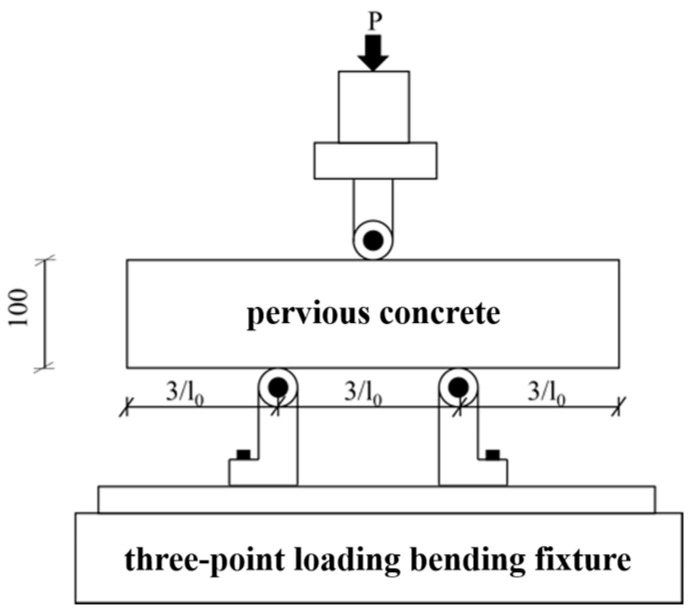
Flexural strength test.

At a curing age of 7 days and 28 days, both carbonated and uncarbonated specimens were taken out of box to determine the compressive strength, according to the Standard Specification for test methods of mechanical properties of ordinary concrete (GB/T 50081-2002) [28]. The flexural strength experiments adopted three-point loading mode, in which the distance between the supports was one-third of the specimen length; the loading method adopted displacement control, and the maximum displacement was controlled to be 10 mm. Diagrammatic sketches of the flexural strength experiments are shown in Figure 1. The mechanical properties of the specimens were tested using a WAW-1000C electro-hydraulic servo universal testing machine. The energy ratio method was used to evaluate the flexural toughness of pervious concrete. According to the American Society for Testing and Materials, the toughness evaluation method of ideal elastoplastic materials (ASTMC1018) [29] was used.

**Figure 2:**
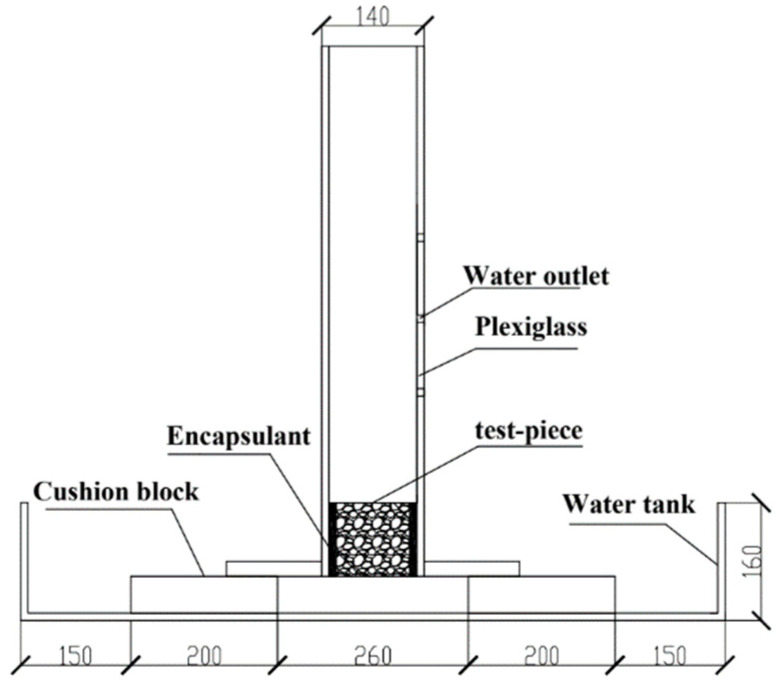
Permeability coefficient constant head test.

The water permeability coefficient of pervious concrete was measured via the constant head method in terms of the Chinese industrial standard Water Permeable Brick (JC/T 945-2005) [30], as shown in Figure 2.

**Figure 3:**
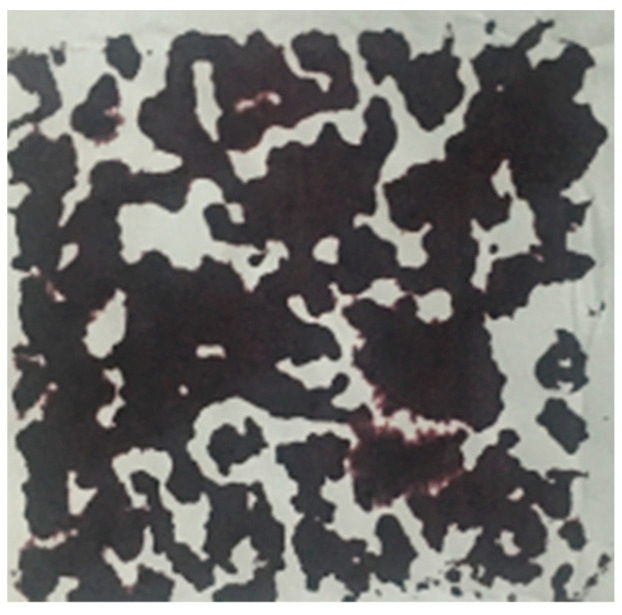
Surface mesoscopic porosity determination method: Ink-printing method.

The effective porosity of pervious concrete is determined according to Technical Specification for Pervious Concrete Pavements (DB11/T 775-2010) [25]. In addition, the micro porosity of the formed surface and bottom surface of permeable concrete specimens was measured using paper cutting and image processing methods to evaluate the plugging and settling conditions of permeable concrete, as shown in Figure 3.

The specimens used to test the degree of carbonization were taken out of the curing room at a curing age of 26 days and baked at 60 °C for 48 h. After drying, one side of the specimen was left to draw parallel lines along the length direction at a spacing of 10 mm as the measurement point for the predetermined carbonization depth. The other surfaces are sealed with heated paraffin. During the test, 1% concentration of phenolphthalein absolute ethanol solution was sprayed on the split surface.

The pervious concrete specimens cured to 28 days were removed. The paste on the surface of the aggregate was scraped with a blade and then ground with an agate mortar to prepare microscopic phase analysis samples. Both X-ray diffraction (XRD) and Fourier Transform Infrared Spectrum (FTIR) were used to determine the phase composition characteristics of the pervious concrete paste. Additionally, the BET analysis technique [31] was used to investigate the specific surface area and micropore structure characteristics of the pervious concrete paste.

## 3. Results and Analysis

### 3.1. Effects of Carbonation on Mechanical Performance

#### 3.1.1. Compressive and Flexural Strength

**Figure 4:**
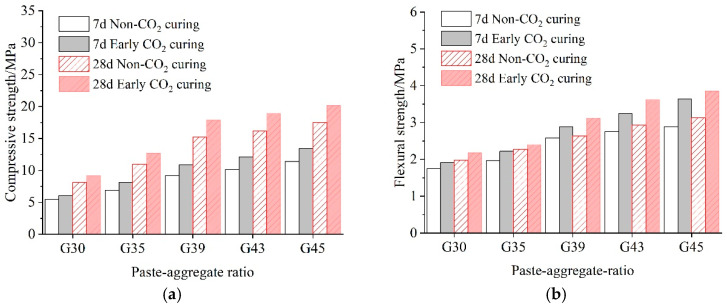
Mechanical strength of the pervious concrete. (**a**) Compressive strength; (**b**) flexural strength.

Figure 4 shows the compressive and flexural strength of pervious concrete under two curing conditions. The number 30 in G30 means the paste–aggregate ratio was 0.3. With the increase in the paste–aggregate ratio and the extension of the curing age, the compressive and flexural strengths of the pervious concrete under both curing conditions show a clear increasing trend. This is consistent with the research conclusions in the literature [32,33]. This is mainly because with the increase in the paste–aggregate ratio, the amount of slurry wrapped in aggregate increases, leading to the increase in strength.

**Figure 5:**
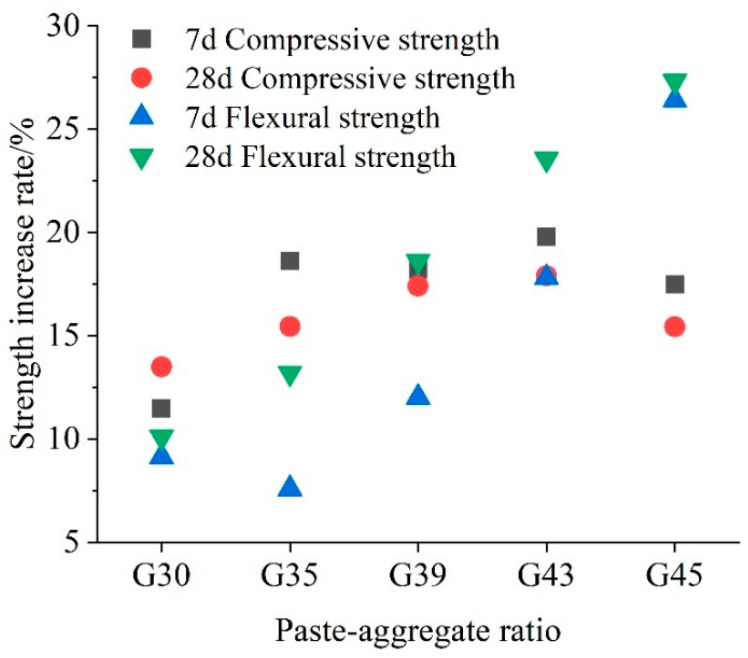
Strength change rate as a function of paste–aggregate ratio caused by early CO_2_ curing.

To further compare the effects of paste–aggregate ratio and curing methods on property, the mechanical strength change rate of 28-day carbonated concrete is calculated and summarized in Figure 5. It can be seen from the figure that in each paste–aggregate ratio, 28-day early CO_2_ curing improved the compressive strength by 13~18% and increased the flexural strength by 5~24%. With the increase in the paste–aggregate ratio, the improvement rate by CO_2_ curing increased first, and then decreased. For flexural strength, the maximum increase rate (23.55%) for early CO_2_ curing occurs with a paste–aggregate ratio of 0.43. This is because the increase in the proportion of paste contributes to the strength development of the pervious concrete matrix, after early CO_2_ curing. However, the maximum increase rate of compressive strength (17.45%) was caused by concrete in a paste–aggregate ratio of 0.39. The increase in the paste–aggregate ratio could improve both flexural strength and compressive strength under two curing conditions, but the positive effect of carbonation becomes less significant when the paste–aggregate ratio increases to a certain value. The phenomenon is more obvious in the comprehensive strength increase rate profiles. In summary, early CO_2_ curing has more significant effects on the development of the flexural strength of pervious concrete, which is consistent with the conclusion in literature that early CO_2_ curing can improve the mechanical properties of concrete [34]. Early CO_2_ curing accelerates the formation of CaCO_3_ and increases the average chain length of calcium silicate hydrate, which is an important reason why early CO_2_ curing can improve the mechanical properties of cement stone. As we all know, flexural strength is an important mechanical property index of pavement structural materials, so this result provides the potential benefits of CO_2_ curing on performance improvement of pervious concrete.

#### 3.1.2. Compressive and Flexural Toughness

**Figure 6:**
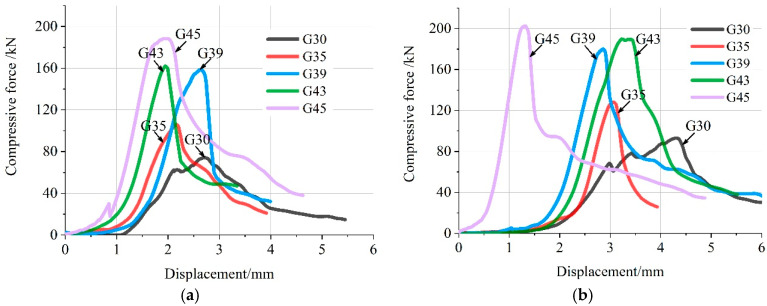
Relations of ultimate failure compressive force and displacement with and without early CO_2_ curing treatment for 28 days. (**a**) Non-CO_2_ Curing. (**b**) Early CO_2_ Curing.

To characterize the toughness of concrete under different conditions, both compressive and flexural force–displacement curves before ultimate failure are averaged and plotted for analysis. Figure 6 presents the results of the ultimate failure compressive load–displacement curve of specimens with and without CO_2_ curing for 28 days. With no early CO_2_ curing treatment, the deformation under ultimate compressive load decreases as the paste–aggregate ratio increases. For example, when the paste–aggregate ratio is 0.30, the corresponding deformation is 4.52 mm. It is about three times that of pervious concrete with a paste–aggregate ratio of 0.45. It can also be seen from Figure 6 that except for the pervious concrete with a paste–aggregate ratio of 0.45, the deformation of other paste–aggregate concrete under the ultimate compressive load under early CO_2_ curing is larger than that without CO_2_ curing, which is consistent with the results in Figure 4. This also indicates that early CO_2_ curing treatment can improve the compressive toughness of pervious concrete to a certain extent.

**Figure 7:**
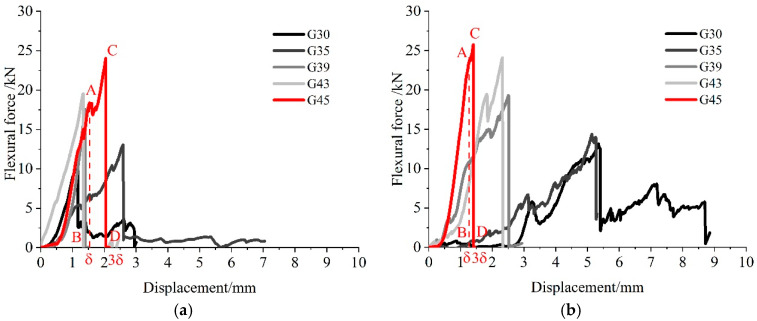
Ultimate flexural load–displacement curves of pervious concrete with and without early CO_2_ curing treatment for 28d. (**a**) Non-CO_2_ Curing. (**b**) Early CO_2_ Curing.

Figure 7 shows the characteristics of the ultimate flexural load–displacement curve of pervious concrete in different paste–aggregate ratios under two curing methods. The flexural toughness index is derived to characterize the toughness. The principle for calculation is as follows. Taking the G45 specimen as an example, the deflection corresponding to the initial crack point B is *δ*, the area enclosed by OAB is *T*_1_; the corresponding abscissa of point D is 3*δ*, and the area enclosed by OACD is *T*_3_. Then, the ratio of the area under the load–displacement curve *T*_3_ to the area under the load deflection curve corresponding to the initial crack point *T*_1_ is defined as the toughness index *I*_3_ to measure the flexural toughness of the pervious concrete, as shown in Formula (1),

(1) $$I_{3}=\frac{T_{3}}{T_{1}}$$

where *T*_1_ and *T*_3_ (N mm) are the areas of OAB and OACD in Figure 7a,b, respectively.

**Figure 8:**
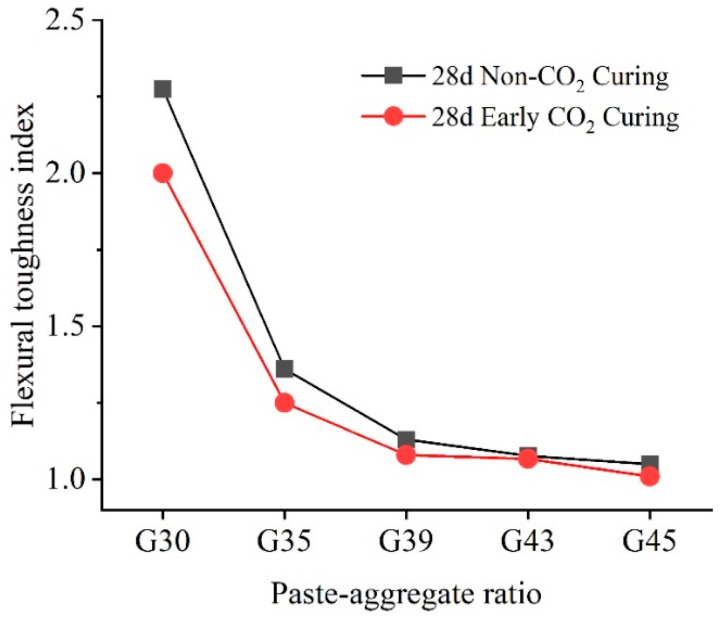
The flexural toughness index of 28-day pervious concrete under two curing conditions in different paste–aggregate ratios.

The final indexes for all specimens at 28 days are calculated and presented in Figure 8. Under two curing conditions, the flexural toughness index gradually decreases with the improvement of the paste–aggregate ratio. The reduction in the buffer area leads to an increase in the brittleness of the structure and a decrease in the toughness. It can also be seen from Figure 8 that the flexural toughness index of the pervious concrete cured by early carbonation is less than that without CO_2_. The difference is mostly sufficient when the paste–aggregate ratio is 0.3. At this ratio, the flexural toughness index of the pervious concrete with early CO_2_ curing treatment is 2.0, which is reduced by 12% compared to pervious concrete without CO_2_ curing (with a flexural toughness index of 2.274). With the increase in the paste–aggregate ratio, the differences in the flexural toughness index between two curing methods become less obvious. When the ratio reaches 0.43, the flexural toughness indexes of concrete under two curing methods are quite close to each other. Therefore, the effects of early CO_2_ curing treatment or flexural toughness is also related to the paste–aggregate ratio.

### 3.2. Analysis of Effective Porosity and Water Permeability

#### 3.2.1. Water Permeability and Effective Porosity

**Figure 9:**
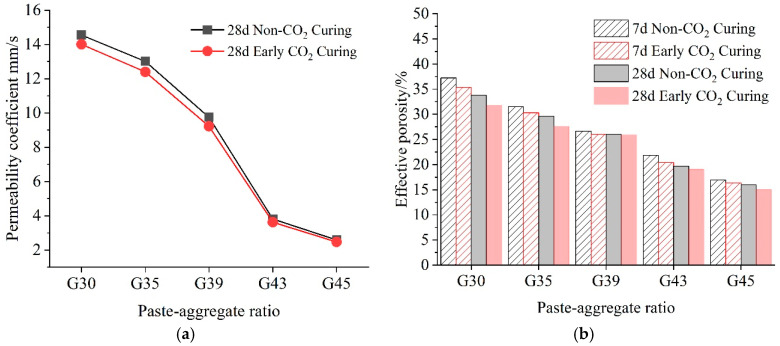
Pervious concrete in different paste–aggregate ratios under two curing conditions. (**a**) Permeability coefficient. (**b**) Effective porosity.

The permeability coefficient mainly indicates the difficulty of fluid passing through the void skeleton [35]. Figure 9a shows the variation in the permeability coefficient of pervious concrete under two curing conditions for 28 days. The water permeability coefficient of the pervious concrete shows a significant decreasing trend with the increase in the paste–aggregate ratio. This is because with the increase in the paste–aggregate ratio, the superfluous slurry began to slip off, resulting in plugging. It can also be seen that under the same paste–aggregate ratio, the permeability coefficient of the pervious concrete with early CO_2_ curing treatment is slightly smaller than that without CO_2_ curing. However, like the pattern in Figure 8, with the increase in the paste–aggregate ratio, the differences in the permeability coefficient under two curing conditions become less significant. After the paste–aggregate ratio exceeds 0.43, the water permeability coefficients become much closer to each other. This trend is consistent with the research results of Zhang [32].

Figure 9b shows the effective porosity of pervious concrete as the paste–aggregate ratio increases under two curing methods. Under two curing methods, with the increase in the paste–aggregate ratio and age, the effective porosity of the pervious concrete shows a decreasing trend, and the effective porosity of the pervious concrete with early CO_2_ curing treatment is slightly lower than that without CO_2_ curing. This is because a large amount of CaCO_3_ formed during carbonation process fills the pore size of cement stone, thus refining its pore structure and making the matrix structure more compact [34], which is consistent with the conclusion of the mechanical analysis. In addition, Zhang believes that the permeability coefficient increases with the increase in effective porosity and presents a Power function relationship [33], which confirms the research results of the test.

#### 3.2.2. Macro-Surface Porosity

**Figure 10:**
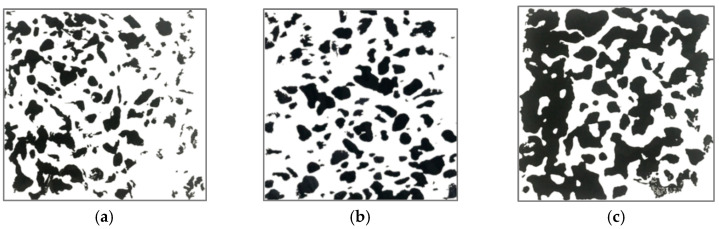

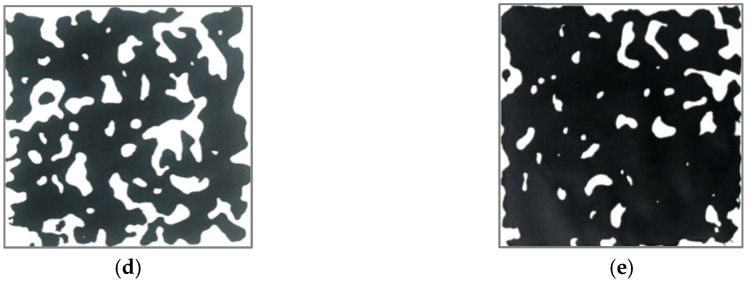
Bottom porous characteristics of porous concrete in different paste–aggregate ratios under early carbonation condition. (**a**) G30; (**b**) G35; (**c**) G39; (**d**) G43; (**e**) G45.

The water resistance of pervious concrete is largely determined by the porosity characteristics. In this part, the effects of curing methods on macro-surface porosity of concrete in different paste–aggregate ratios are analyzed. Figure 10 presents the image processing results of the bottom surface of pervious concrete in different paste–aggregate ratios under the early CO_2_ curing condition. The black colors refer to concrete, and the white gap in the image reflects the pores. As shown in this figure, as the paste–aggregate ratio increases, the bottom pore of the pervious concrete decreases significantly. When the paste–aggregate ratio reaches 0.43, obvious sedimentation of the paste occurs.

**Figure 11:**
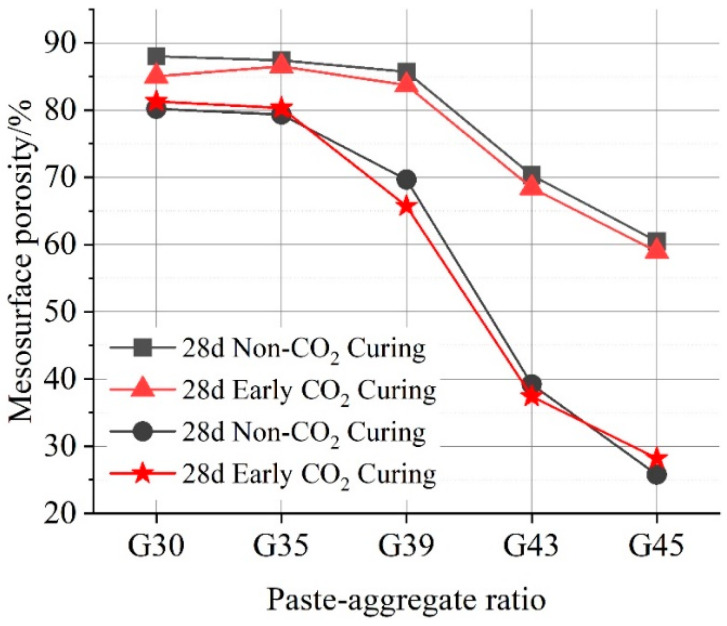
The relationship between macroscopic porosity of pervious concrete surface in different paste–aggregate ratios under two curing conditions.

Based on the area covered by white gaps in the image, the macro-surface porosity of 28-day pervious concrete in different paste–aggregate ratios is calculated and listed in Figure 11. Porosity calculated with both top and bottom surface of the concrete is plotted. With the increase in the paste–aggregate ratio, the macro-surface porosity of pervious concrete with and without early CO_2_ curing treatment shows a decreasing trend. The porosity on the top surface is larger than that on the bottom surface. Besides, the micro-surface porosity under early CO_2_ curing condition is slightly lower than that under no CO_2_ curing conditions. Therefore, early CO_2_ curing treatment can to some extent refine the micro-surface property of pervious concrete, particularly when the paste–aggregate ratio is lower than 0.43.

### 3.3. Carbonation Degree

**Figure 12:**
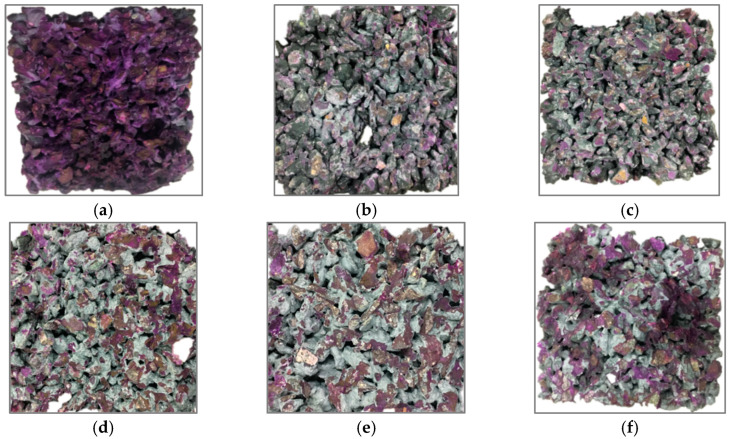
Carbonation degree of pervious concrete in different paste–aggregate ratios under early CO_2_ curing condition for 28 days. (**a**) Uncarbonated concrete; (**b**) carbonated G30; (**c**) carbonated G35; (**d**) carbonated G39; (**e**) carbonated G43; (**f**) carbonated G45.

The carbonization degree of each pervious concrete specimen is shown in Figure 12. Figure 12a gives an example of concrete with no CO_2_ curing. After spraying the phenolphthalein solution, the entire surface is a red color. Therefore, by comparing the area with and without red color, the carbonation degree of pervious concrete can be derived.

Figure 12b–f presents the surface of concrete in different paste–aggregate ratios under early CO_2_ curing for 28 days. As shown in Figure 12b, the split surface of the pervious concrete with a paste–aggregate ratio of 0.30 is basically colorless. It shows that the slurry has basically been completely carbonized. As shown in Figure 12c, the center of the split surface shows a small amount of dark red, and the outer circle is colorless, indicating that the internal slurry is not completely carbonized. As shown in Figure 12d,e, the dark red color is expanding outward, and the colorless paste is decreasing. As shown in Figure 12f, when the paste–aggregate ratio is 0.45, there are a lot of dark red parts on the splitting surface of pervious concrete, and the outer part of the slurry is colorless. This indicates that the carbonization degree of pervious concrete is quite low. This is basically consistent with Chen’s discovery that the higher the porosity, the more conducive it is to the internal diffusion of gaseous CO_2_ [15].

Summarizing from Figure 12, under the early CO_2_ curing condition, the carbonization degree of the pervious concrete gradually decreases with the increase in the paste–aggregate ratio. The possible reason is that as the paste–aggregate ratio increases, the larger amount of slurry filled between the aggregates increases. Meanwhile, when the ratio of paste aggregate is further increased, the excess slurry slips to the bottom of the specimen, and the phenomenon of sedimentation occurs. In other words, the speed of carbon dioxide entering the specimen slows down, resulting in a decrease in the carbonation degree. This phenomenon explains the less significant effects of carbonation on porosity when the paste–aggregate ratios reach 0.39.

### 3.4. Effects on Microstructure and Mechanism Analysis

#### 3.4.1. Microscopic Morphology

**Figure 13:**
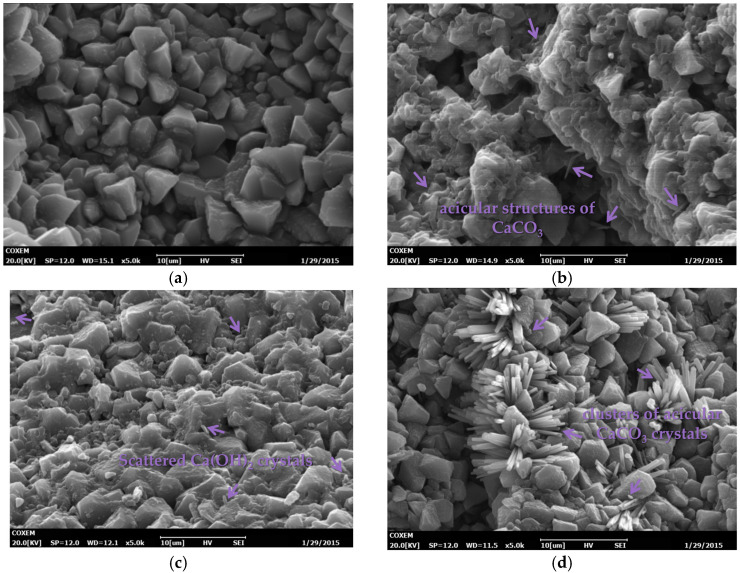
SEM micrograph of pervious concrete paste with early CO_2_ curing treatment for 28 days. (**a**) Uncarbonized G39; (**b**) carbonated G39; (**c**) carbonated G35; (**d**) carbonated G45.

To analyze the mechanism with microscopic perspective, the SEM electron microscope was used to characterize the microstructure morphology of 28-day aggregate surface slurry under two curing methods. In particular, the Ca(OH)_2_ crystal enrichment site was selected to compare the changes in hydration products under two curing conditions. Figure 13a presents the pervious concrete slurry cured without CO_2_ for 28 days, which mostly manifested as hexagonal layered calcium hydroxide crystals, with a small amount of gel attached to the surface of the crystals. The adhesion between the crystals was relatively weak. Chen’s research also mentioned relevant conclusions [19].

Figure 13b–d compares the SEM results of carbonated pervious concrete paste in different paste–aggregate ratios. As shown in Figure 13b, the surface of the hexagonal layered calcium hydroxide crystals is uniformly covered with a large amount of gel, and the crystals are tightly bonded, showing better integrity. There is a good deal of acicular structures of CaCO_3_, as shown by the arrows in the figure, and the structural strength is further improved. Figure 13c is the microscopic appearance of the pervious concrete slurry when the paste–aggregate ratio is 0.35. It can be seen that the calcium hydroxide crystals of the layered structure are scattered, and the arrangement is not compact, and there is a small amount of acicular CaCO_3_ in the slurry. Figure 13d shows the microscopic appearance of the 28-day pervious concrete slurry with a paste–aggregate ratio of 0.45. The hexagonal layered calcium hydroxide crystals are tightly distributed, and numerous clusters of acicular CaCO_3_ crystals appear between the calcium hydroxide crystals, indicating that the structure has been further improved.

#### 3.4.2. Phase Composition

**Figure 14:**
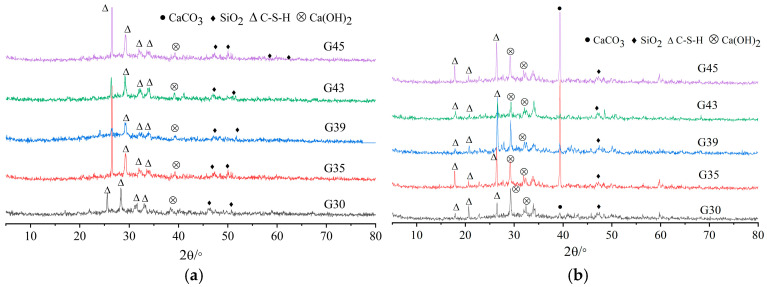
XRD pattern of pervious concrete paste curing for 28 days with and without early CO_2_ curing treatment. (**a**) Non-CO_2_ curing; (**b**) early CO_2_ Curing.

The XRD phase compositions of the pervious concrete slurry with and without early CO_2_ curing treatment for 28 days are shown in Figure 14. The main peak of CaCO_3_ of pervious concrete slurry under early CO_2_ curing condition is much larger than that without CO_2_ curing, while the main peak of calcium hydroxide crystal is smaller than that without CO_2_ curing. This is because the early CO_2_ curing treatment speeds up the reaction between CaO and CO_2_, generating more CaCO_3_. However, the C-S-H gel content in pervious concrete slurry under the early carbonation is lower than that without CO_2_ curing. Figure 14 also shows that as the paste–aggregate ratio increases, the overall calcium hydroxide crystal peak increases, while the CaCO_3_ peak first increases and then decreases. This is because as the paste–aggregate ratio and the slurry content increase, the internal slurry carbonation degree is less significant, which inhibits the formation of CaCO_3_.

**Figure 15:**
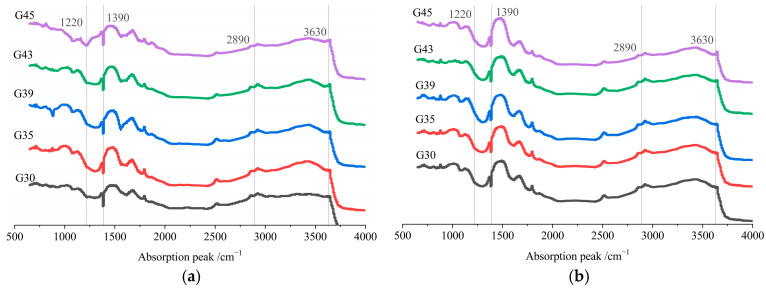
FTIR spectrum of pervious concrete paste after 28 days with and without early CO_2_ curing treatment. (**a**) Non-CO_2_ Curing; (**b**) early CO_2_ curing.

The FTIR spectrums of pervious concrete slurry with and without early CO_2_ curing treatment for 28 days are shown in Figure 15. The absorption peak at 3630 cm^−1^ is the stretching vibration of OH in Ca(OH)_2_. According to Figure 15b, the absorption peak near 2890 cm^−1^ disappears, indicating that Ca(OH)_2_ reacts with carbon dioxide. The absorption peaks near 1390 cm^−1^ and 1220 cm^−1^ are the antisymmetric stretching vibration and plane bending vibration of CO_3_^2−^ in calcite, which indicates that calcite is formed by the reaction between Ca(OH)_2_ and carbon oxide. Compared with the IR spectrum of paste cured without CO_2_, the shift frequency of IR spectrum cured by CO_2_ is about 20 cm^−1^. The blue shift of the absorption band indicates that the calcite crystal increases; that is, the CaCO_3_ content in the specimen increases.

In summary, in the early carbonation process, the hydrated calcium silicate in the hydrated product was gradually decomposed to form CaCO_3_. The CaCO_3_ that appeared during the carbonation process was mainly in the form of calcite. When CaCO_3_ is formed, SiO_2_ gel is also produced. When it is fully carbonized, due to the complete decomposition of calcium silicate hydrate, there are only characteristic absorption peaks of SiO_2_ gel and CaCO_3_ in the IR spectrum [19].

#### 3.4.3. Microscopic Pore Structure

**Figure 16:**
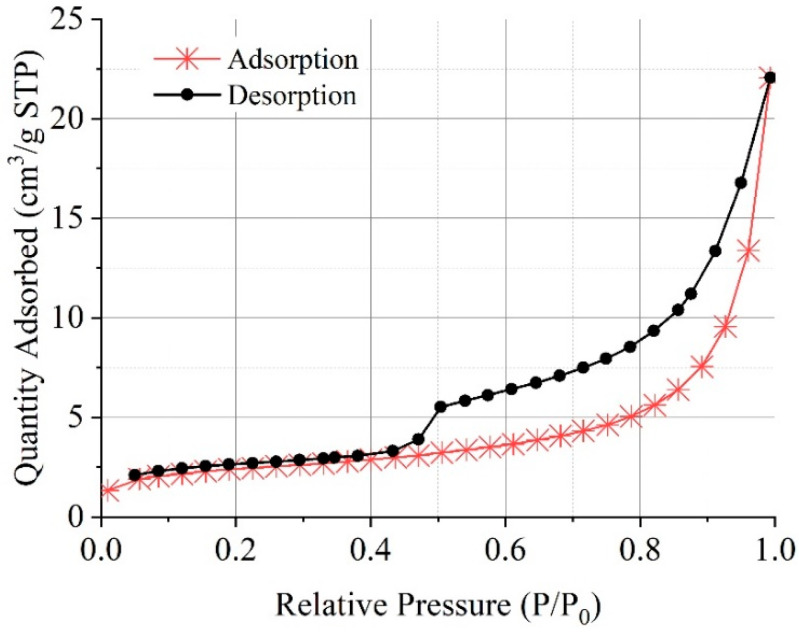
Absorption and desorption isotherms of pervious concrete slurry under early CO_2_ curing condition.

To analyze the effects on pore structure of pervious concrete at the microscopic perspective, the BET theory is applied to characterize the microscopic pore. The adsorbate in Bet pore distribution analysis adopts nitrogen adsorption, and the temperature of the analysis tank is 195–850 °C, while the heating rate is 10 °C/min. The adsorption and desorption isotherms are shown in Figure 16. According to the Langmuir adsorption isotherm equation [36], the Bet adsorption isotherm equation is derived as,

(2) $$\frac{P}{V(P_{0}-P)}=\frac{1}{V_{m}C}+\frac{C-1}{V_{m}C}\cdot \frac{P}{P_{0}}$$

where *P*_0_ is the saturated vapor pressure of the adsorbate at the adsorption temperature; *V_m_* is the saturated adsorption capacity of the monolayer; C is the Bet equation constant, its value is exp{(E_1_ − E_2_)/RT}, where E_1_ is the adsorption heat of the first adsorption layer.

It can be seen from Formula (2) that when the experimental data of physical adsorption *P*/*V*(*P*_0_ − *P*) and *P*_0_/*P* are plotted, a straight line is obtained. The slope of the straight line is m = (*C* − 1)/(*V_m_C*), and the intercept on the vertical axis is b = 1/(*CV_m_*), where *V_m_*, i.e., the saturated adsorption capacity of the single layer can be calculated as

(3) $$V_{m}=\frac{1}{m+b}$$

Assuming that the average cross-sectional area of each adsorbed molecule is *A_m_*(nm^2^), this *A_m_* is the surface area occupied by the adsorbent molecule on the surface of the adsorbent

(4) $$S_{g}=A_{m}N_{A}\cdot \frac{V_{m}}{22414}\times 10^{-18}$$

where *N_A_* is Avogadro’s constant (6.02 × 10^23^); S_g_ is the specific surface area (m^2^/g). Emmet and Brownauer [37] have proposed that the cross-section of the liquid hexagonal close-packed nitrogen molecule at 77 K (−195 °C) is 0.162 nm^2^, so Formula (3) can be simplified to obtain the Bet nitrogen adsorption specific surface area as

(5) $$S_{g}=4.325V_{m}$$

**Figure 17:**
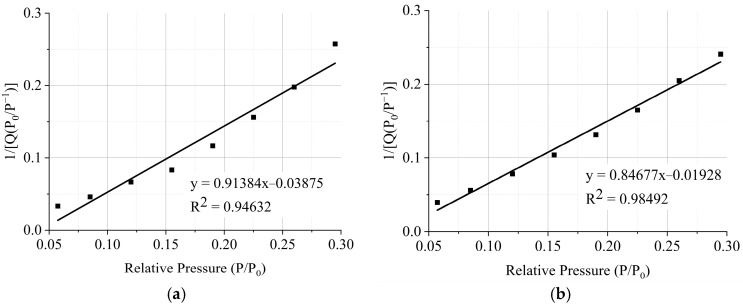
Determination of specific surface area of pervious concrete paste under early CO_2_ curing condition. (**a**) Non-CO_2_ curing; (**b**) early CO_2_ curing.

**Figure 18:**
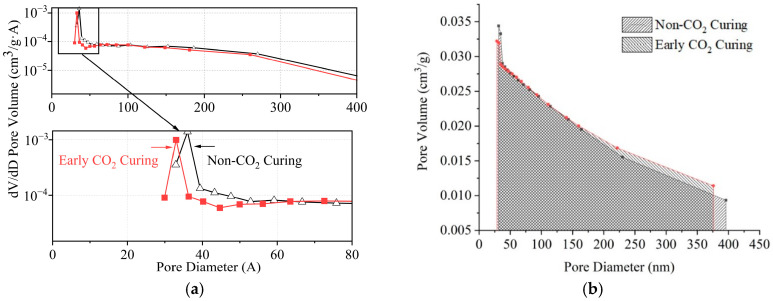
Bet pore size distribution of pervious concrete paste under early CO_2_ curing condition. (**a**) dV/dD pore volume; (**b**) cumulative pore volume.

Through the principles above, the specific surface area of the pervious concrete slurry is measured by calculating the slope and intercept of the straight line, as shown in Figure 17. After linear fitting, R^2^ meets the experimental requirements. The specific surface area of pervious concrete slurry cured by early carbonization is 5.25 cm^2^/g, which is 5.85% higher than that cured without carbonation. This is because with early CO_2_ curing treatment, CO_2_ directly reacts with C-S-H gel and the generated CaCO_3_ mainly exists in the form of calcite; the calcium–silicate ratio of C-S-H gel decreases, and the specific surface area increases after carbonization. The Barrett–Joyner–Halenda model was adopted to further analyze the pore size distribution. The basic principle of the model is introduced in the literature [38]. The results are presented in Figure 18. The early CO_2_ curing treatment will reduce the micropores with a pore size less than 40 nm, and the mesopores with a pore size greater than 40 nm will increase, but the overall pore volume is reduced with early CO_2_ curing treatment.

## 4. Conclusions

To improve the comprehensive performance of pervious concrete, this study compared the mechanical properties, water permeability, porosity, and chemical composition of pervious concrete under different paste–aggregate ratios and two curing conditions. Below, we summarize the main conclusions of this paper based on the aforesaid findings:

(1) With the increase in the paste–aggregate ratio and curing time, the mechanical properties of pervious concrete are improved to some extent under both curing conditions. In addition, the mechanical properties of pervious concrete after early CO_2_ curing are higher than those without CO_2_ curing at all paste–aggregate ratios.
(2) Early CO_2_ curing can improve the compressive toughness of pervious concrete but reduces its flexural toughness. However, its influence on flexural toughness decreases with the increase in the paste–aggregate ratio.
(3) The permeability and effective porosity of pervious concrete decrease significantly with the increase in the paste–aggregate ratio. Additionally, pervious concrete cured with early carbonization has poor water permeability and smaller porosity due to its denser structure.
(4) Under early CO_2_ curing conditions, the degree of carbonization in pervious concrete gradually decreases with the increase in the paste–aggregate ratio.
(5) In the research on micro mechanisms, it was found that CO_2_ in pervious concrete directly reacts with C-S-H gel under early CO_2_ curing treatment, and CaCO_3_ mainly exists in the form of calcite. With the increase in the paste–aggregate ratio, the formation of CaCO_3_ is inhibited due to the decrease in carbonation degree in pervious concrete. At the same time, early CO_2_ curing condition will reduce the pore size by less than 40 nm in pervious concrete and increase the pore size by greater than 40 nm. It is worth mentioning that the overall pore volume is greater in pervious concrete.

The results indicate that early CO_2_ curing can help improve the comprehensive performance of pervious concrete, which has guiding significance for the preparation of high-performance pervious concrete and the construction of the ecological environment. In any case, further research is required to quantify and improve the carbonation degree of pervious concrete in different paste–aggregate ratios, which is the focus of our future research content.

## Figures and Tables

**Table 1:** Chemical composition of cement P·O42.5.

Mass Fraction (%)						
**SiO**_**2**_	**Al**_**2**_ **O**_**3**_	**Fe**_**2**_ **O**_**3**_	**CaO**	**MgO**	**SO**_**3**_	**Others**
16.23	2.81	4.30	69.52	1.33	4.17	1.64

**Table 2:** Performance parameters for cement P·O42.5.

Density (g/cm^3^)	Flexural Strength (MPa)		Compressive Strength (MPa)	
	3d	28d	3d	28d
3.3	5.73	11.48	26.86	44.2

**Table 3:** Physical index of aggregate.

Aggregate Gradation (mm)	Apparent Density $\rho_{A}$ (kg/m^3^)	Compacted Bulk Density $\rho_{G}$ (kg/m^3^)	Specific Surface Area (Quality) S_1_ (cm^2^/g)	Percentage of Mud Content (%)
4.75–9.5	2648	1470	2.99	<1

**Table 4:** Mix proportion of pervious concrete (kg/m^3^).

No.	Paste–Aggregate Ratio	Aggregate	P·O42.5	Water	Superplasticizer
G45 ^a^	0.45	1512	494.71	98.94	9.89
G43 ^a^	0.43	1476	472.72	94.54	9.45
G39 ^a^	0.39	1400	438.11	87.62	8.76
G35 ^a^	0.35	1355	396.15	79.23	7.92
G30 ^a^	0.30	1307	337.54	67.51	6.75

^a^ The number XX indicates the ratio of the paste–aggregate, i.e., the paste–aggregate ratio is 0.XX%.

## Data Availability

No data was used for the research described in the article.
